# STOP Codon Mutations at Sites of Natural Caspase Cleavage Are Implicated in Autism and Alzheimer’s Disease: The Case of ADNP

**DOI:** 10.3389/fendo.2022.867442

**Published:** 2022-03-23

**Authors:** Illana Gozes, Shula Shazman

**Affiliations:** ^1^ Elton Laboratory for Molecular Neuroendocrinology, Department of Human Molecular Genetics and Biochemistry, Sackler Faculty of Medicine, Adams Super Center for Brain Studies and Sagol School of Neuroscience, Tel Aviv University, Tel Aviv, Israel; ^2^ Department of Mathematics and Computer Science, The Open University of Israel, Raanana, Israel

**Keywords:** apoptosis, activity dependent neuroprotective protein (ADNP), caspase, mutations, autism spectrum disorder, Alzheimer’s disease

## Introduction

Activity-dependent neuroprotective protein (ADNP) (1, 2) was originally discovered at the Gozes laboratory as a glial secreted protein, in the presence of the G-protein-coupled receptors (GPCR)-neuropeptide activator, vasoactive intestinal peptide (VIP) (1). With pituitary adenylate cyclase (PACAP) exhibiting extensive homology to VIP, later studies identified PACAP regulation of ADNP (3–7). Essential for brain development and function (8, 9), ADNP is identified as one of the leading *de novo* mutated gene causal for an autism/intellectual disability syndrome, the ADNP syndrome (also known as Helsmoortel Van Der Aa) (10–12). Furthermore, recent studies in the Gozes laboratory identified somatic mutations in ADNP in Alzheimer’s disease brains correlating with the progression of Tau pathology (13), and paralleled by Tau depositions in the ADNP syndrome young postmortem brain (14). ADNP functions as a microtubule regulator, enhancing Tau-microtubule binding and protecting against Tauopathy (15). ADNP also functions as a chromatin remodeler (16), further involved in alternative splicing (17) and DNA damage repair (18), regulating thousands of proteins (19). As such, it is our opinion that ADNP is central to key cellular processes. Thus, paralleling disease inflicting truncating mutations in ADNP to natural protein cleavage sites will identify basic disease – related cellular mechanisms, leading to better disease management.

## Similarity in ADNP Length After Cleavage by Proteases or Truncation by ADNP Mutations

**Figure 1:**
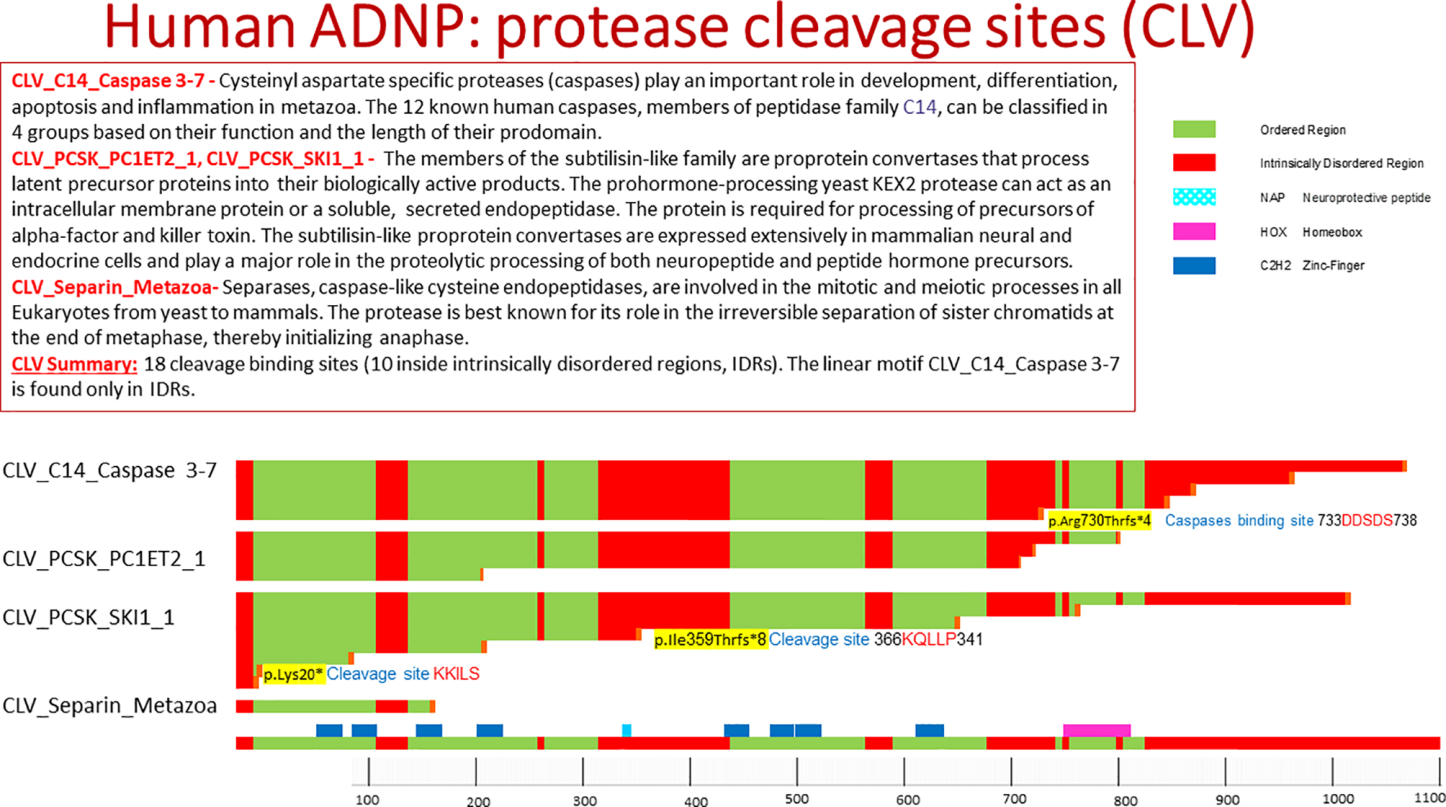
Human ADNP protease cleavage site (CLV). The picture shows protein cleavage sites in ADNP as obtained from the ELM data base. Highlighted in the text are cleavage sites associated with somatic ADNP mutations in postmortem Alzheimer’s disease brains. The ELM site shows ordered and disordered structures within protein, with ordered meaning a three dimensional protein structure.

Computational analysis by the eukaryotic linear motif (ELM) prediction tool (20) identified ADNP cleavage sites including the following classes (**Figure 1**).

### Cysteinyl Aspartate Specific Proteases (Caspases)

Caspases (21) represent key players in apoptosis, development and differentiation. Caspases recognize the respective substrates by specific cleavage motifs. There are five amino acids of the substrate around the caspase cleavage site, named (N- to C-terminal): P4, P3, P2, P1, P-1. The scissile bond between the essential aspartate at P1 and P-1, usually a small amino acid, is cleaved by caspase-3 and -7, whereas positions P4 to P-1 are important for substrate specificity and recognition. ADNP residues 734-738 contain the motif DDSDS which is a recognition motif for caspase-3 and caspase-7. As indicated above, cleavage of the caspase substrates results in characteristic morphological features of apoptotic cell death, including membrane blebbing, pyknotic nuclei, cell rounding, and formation of apoptotic vesicles. Thus, activated caspase-3, a major enzyme in the apoptotic pathway, is often used as a marker for apoptotic cells. The length of the ADNP protein after the caspase cleavage is 737aa. Interestingly, one of the most prevalent autism/intellectual disability causing *de novo* mutations in ADNP in p.Arg730* (10), closely located near the caspase cleavage site. Furthermore the recurrent somatic ADNP frameshift mutation p.Arg730Thrfs*4, which is one of the pathogenic mutations in ADNP that is correlated to aging/Alzheimer’s disease, truncates ADNP length to a protein of 734aa (13) (**Figure 1**).

### CLV_PCSK_SKI1_1 (http://elm.eu.org/elms/CLV_PCSK_SKI1_1.html)

The subtilisin-like proprotein convertases (PCSKs) mammalian subtilisin/kexin isozymes (SKIs) are expressed extensively in mammalian neural and endocrine cells and play major roles in the proteolytic processing of both neuropeptides and hormone precursors. The members of the subtilisin-like family are proprotein convertases that process latent precursor proteins into biologically active products. PCSK1 (proprotein convertase 1, NEC1) and PCSK2 (proprotein convertase 2, NEC2) are type I proinsulin-processing enzymes important in regulating insulin biosynthesis. These enzymes also cleave (for example) proopiomelanocortin, prorenin, proenkephalin, prodynorphin, prosomatostatin and progastrin (22–24).

ADNP residues 367-371 KQLLP include the cleavage motif of recognized by the members of the subtilisin-like family. The length of ADNP after cleavage in this site is 367aa. Interestingly, the length of ADNP after the truncating mutation p.Ile359Thrfs*8 is 367aa, found in the post mortem Alzheimer’s brain (13).

PACE4 (paired basic amino acid cleaving system 4, SPC4) is a calcium-dependent serine endoprotease that can cleave precursor protein at paired basic amino acid processing sites [e.g. p.Lys20*, **Figure 1**, found in the postmortem Alzheimer’s brain (13)]. Its substrates include transforming growth factor beta-related proteins, proalbumin, and von Willebrand factor and assorted neuropeptides. Several paired basic amino acids are found in ADNP (**Figure 1**).

## Discussion

The discovery of ADNP included the identification of a short active motif within ADNP, termed NAP (NAPVSIPQ, **Figure 1**) (1). NAP enhances ADNP binding to microtubule end binding protein (EB1 and EB3) (25), in turn augmenting Tau microtubule interactions, protecting against tauopathy, even in the face of ADNP mutations, such as the prevalent mutation, p.Arg730* (13–15). Further studies have shown that NAP protects against activated caspase 3 associated apoptosis (26–28). Caspase 3 activation is mediated by cytochrome C (29), protected by NAP (30), whereas cytochrome C is released from mitochondria is enhanced by p53 (31) and ADNP/NAP reduce p53 (2, 32, 33). In turn, DNA damage results in posttranslational modifications of p53 (34), activating the release of cytochrome C (29).

Regarding DNA damage, R-loops are three-stranded nucleic acid structures that accumulate on chromatin in neurological diseases and cancers and contribute to genome instability. ADNP resolves/suppresses R-loops. Importantly, deletion of the ADNP homeodomain severely diminishes R-loop resolution activity, compromising neuronal differentiation. Additionally, patient-derived human induced pluripotent stem cells that contain the prevalent ADNP syndrome-causing mutation p719* exhibit R-loop and CTCF accumulation at ADNP targets (18, 35). These findings, together with our current bioinformatics observations suggest that ADNP cleavage by caspase 3, may be deleterious at two levels: 1] enhancing DNA damage, and 2] reducing ADNP-Tau-microtubule interactions, resulting in tauopathy and followed or paralleled by apoptosis. These findings implicate ADNP as part of the apoptotic pathways in neuronal cells.

Furthermore, Bend et al., identified two distinct and partially opposing genomic DNA methylation episignatures in the peripheral blood samples from 22 patients with ADNP syndrome. The “epi-ADNP-1” episignature included ~ 6000 mostly hypomethylated CpGs, and the “epi-ADNP-2” episignature included ~ 1000 predominantly hypermethylated CpGs. The two signatures correlated with the locations of the ADNP mutations. Epi-ADNP-1 mutations occupy the N- and C-terminus, and epi-ADNP-2 mutations are aggregated on the nuclear localization signal (36). These findings suggest epigenetic activities to the different ADNP cleaved fragments.

Also interesting and related are the findings of caspase 3 - dependent proteolytic cleavage of Tau causes neurofibrillary tangles and results in cognitive impairment during normal aging (37). This is coupled with the finding of plasma P-tau217 levels increasing during the early preclinical stages of Alzheimer’s disease when insoluble tau aggregates are not yet detectable by tau-positron emission tomography (PET), presenting an early biomarker (38, 39). However, an even earlier biomarker is suggested in an unfolded conformational variant of p53, apparent at least 6 years prior to disease onset (40). Phosphorylated Tau and modified p53 in prodromal Alzheimer’s disease are also associated with ADNP found to be the only protein decreasing in Alzheimer’s disease patients’ serum samples (41) and with ADNP serum levels correlating with intelligence, in cognitively intact healthy elderly (42). Importantly, ADNP indirectly interacts with sirtuin 1 (SIRT1) at the chromatin and microtubule/Tau levels (43) as well as regulates Forkhead box O3 (FOXO3) (11), two important genes associated with healthy aging.

Taken together, our studies suggest ADNP directed therapy in susceptible individuals exhibiting the modified p53 biomarker. An ideal therapy would be nasal NAP (davunetide) administration with previous human experience and cognitive score protection/enhancement in amnestic mild cognitive impairment patients (44, 45).

## Author Contributions

IG orchestrated the project and wrote the paper. SS identified the cleavage sites and performed the bioinformatics. Both authors contributed to the article and approved the submitted version.

## Funding

IG is supported by ERA-NET neuron ADNPinMED, as well as Drs. Ronith and Armand Stemmer (French Friends of Tel Aviv University), Holly and Jonathan Strelzik (American Friends of Tel Aviv University) and - Anne and Alex Cohen (Canadian Friends of Tel Aviv University).

## Conflict of Interest

NAP (davunetide) is under patent protection for clinical use. ADNP is under patent protection for Alzheimer’s disease diagnosis (IG).

The remaining author declares that the research was conducted in the absence of any commercial or financial relationships that could be construed as a potential conflict of interest.

## Publisher’s Note

All claims expressed in this article are solely those of the authors and do not necessarily represent those of their affiliated organizations, or those of the publisher, the editors and the reviewers. Any product that may be evaluated in this article, or claim that may be made by its manufacturer, is not guaranteed or endorsed by the publisher.
